# Assessing Tumor Response to Treatment in Patients with Lung Cancer Using Dynamic Contrast-Enhanced CT

**DOI:** 10.3390/diagnostics6030028

**Published:** 2016-07-21

**Authors:** Louise S. Strauch, Rie Ø. Eriksen, Michael Sandgaard, Thomas S. Kristensen, Michael B. Nielsen, Carsten A. Lauridsen

**Affiliations:** 1 Department of Diagnostic Radiology, Copenhagen University Hospital, Rigshospitalet, 2100 Copenhagen, Denmark; roestbjerge@gmail.com (R.Ø.E.); michael@sandgaard.org (M.S.); tskaarup@yahoo.com (T.S.K.); mbn@dadlnet.dk (M.B.N.); cala@phmetropol.dk (C.A.L.); 2 Department of Technology, Faculty of Health and Technology, Metropolitan University College, 2200 Copenhagen, Denmark

**Keywords:** DCE-CT, Dynamic Contrast-Enhanced CT, lung cancer, treatment response

## Abstract

The aim of this study was to provide an overview of the literature available on dynamic contrast-enhanced computed tomography (DCE-CT) as a tool to evaluate treatment response in patients with lung cancer. This systematic review was compiled according to Preferred Reporting Items for Systematic Reviews and Meta-Analyses (PRISMA) guidelines. Only original research articles concerning treatment response in patients with lung cancer assessed with DCE-CT were included. To assess the validity of each study we implemented Quality Assessment of Diagnostic Accuracy Studies (QUADAS-2). The initial search yielded 651 publications, and 16 articles were included in this study. The articles were divided into groups of treatment. In studies where patients were treated with systemic chemotherapy with or without anti-angiogenic drugs, four out of the seven studies found a significant decrease in permeability after treatment. Four out of five studies that measured blood flow post anti-angiogenic treatments found that blood flow was significantly decreased. DCE-CT may be a useful tool in assessing treatment response in patients with lung cancer. It seems that particularly permeability and blood flow are important perfusion values for predicting treatment outcome. However, the heterogeneity in scan protocols, scan parameters, and time between scans makes it difficult to compare the included studies.

## 1. Introduction

Lung cancer is the leading cause of cancer death in males and the second leading cause in females next to breast cancer [1]. Despite improvements in treatment, the five-year survival rate is still low at 18% in patients with lung cancer in the USA [2]. Chances of survival are highly dependent on early detection, but most symptoms do not appear until the disease is already at an advanced stage [3]. Thus, early detection and early assessment of treatment response is essential.

Chemotherapy combined with anti-angiogenic drugs has shown great potential for patients with advanced lung cancer [4,5]. With the introduction of anti-angiogenic drugs, Response Evaluation Criteria in Solid Tumors (RECIST) may be inadequate to evaluate tumor response to treatment. RECIST classifies patients as responders or non-responders primarily due to changes in tumor size [6]. However, anti-angiogenic drugs inhibit tumor growth and progression rather than causing tumor regression. Therefore, new functional imaging techniques aimed at measuring changes in vascular patterns are required [7].

Different functional imaging modalities—such as computed tomography (CT), magnetic resonance imaging (MRI), and ultrasound—have shown promising noninvasive ways to determine changes in tumor vascularity [8]. In particular, dynamic contrast-enhanced CT (DCE-CT) is able to assess the vascular support of tumors by analyzing the temporal changes in attenuation of blood vessels and tissues [9,10]. Furthermore, DCE-CT has the advantages of widespread availability and relatively low cost in addition to high spatial resolution [11]. DCE-CT is already a well-established tool used for assessing acute stroke and is showing great potential in oncology imaging [12]. The aim of this study was to provide a systematic overview of the literature available on DCE-CT used to evaluate early treatment response in patients with lung cancer.

## 2. Materials and Methods 

This systematic review was compiled according to Preferred Reporting Items for Systematic Reviews and Meta-Analyses (PRISMA) 2009 guidelines [13]. 

The literature search was performed in PubMed, Embase, Web of Science, and Cochrane Library on 11 April 2016. Using PubMed as an example, we combined our search of relevant search terms. To include studies that were not yet given a Medical Subject Heading (MeSH) term, we combined MeSH terms with free text search. 

The combination of search word, relevant to our aim of this study, resulted in the following algorithm: 

  **PubMed Search String**  *Tomography, X-ray Computed [MeSH Terms] OR CT OR “Computed Tomography”*  *AND*  *Perfusion imaging [MeSH Terms] OR Perfusion OR Dynamic OR DCE-CT OR “Dynamic contrast-enhanced”*
  *AND*  *Lung Neoplasms [MeSH Terms] OR “Lung cancer” OR “Lung Neoplasms” OR “Non Small Cell Lung Cancer” OR NSCLC*

The search was limited to studies in English, which were published within the last 10 years (2006–2015) to include the most recent research.

After removal of duplicates, all studies included in the search result were screened by title and abstract by two authors (L.S.S. and C.A.L.). Only original research articles concerning treatment response in patients with lung cancer measured with DCE-CT scans were included. All included articles were subsequently retrieved and read by the same two authors. Consensus was reached through discussion. All reference lists of the included articles were searched manually for further references. 

We registered author, publication year, study design, number of participants, diagnosis of the participants, scan parameters, kinetic model, aim, treatment, time of scanning, DCE-CT values, gold standard, outcome, and conclusion for all included studies. To assess the validity of each study we implemented Quality Assessment of Diagnostic Accuracy Studies (QUADAS-2) tool [14]. The tool comprises four domains: patient selection, index test, reference standard, and flow and timing. Each domain is assessed in terms of risk of bias, and the first three domains are also assessed regarding applicability.

## 3. Results

### 3.1. Study Selection and Overview

**Figure 1:**
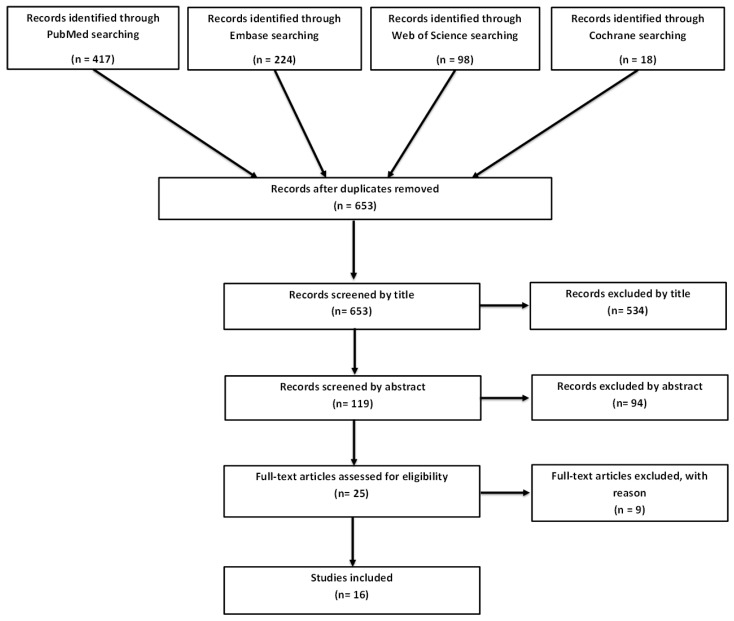
Flow diagram according to Preferred Reporting Items for Systematic Reviews and Meta-Analyses (PRISMA).

After removal of duplicates, the initial search yielded 653 publications. Five-hundred-thirty-four articles were excluded by title, and further 94 were excluded by abstract. Of the remaining 25 articles that were screened by full-text, 16 were included in this study. The study selection is summarized in Figure 1. 

The articles were divided into groups according to treatment as shown in Table 1 and Table 2. Of the 16 articles, seven used systemic chemotherapy alone or combined with anti-angiogenic drugs as treatment (Table 1). In three studies, the patients were treated with radiotherapy, in one study patients were treated with chemotherapy, radiotherapy, or concurrent chemoradiotherapy. Treatments used in the last five articles were thermotherapy, intra-arterial chemotherapy, or target therapy, such as recombinant human endostatin (RHES), anti-epidermal growth factor receptor (EGFR), or anti-angiogenic drugs. These were categorized as others (Table 2).

Most articles used RECIST as gold standard, but four articles had no available gold standard [15,16,17,18]. Lind et al. referred to RECIST and Crabb, which is a tumor evaluation tool for cavitating lesions that subtracts the longest diameter of cavitation from the longest diameter of the lesion [19]. 

Jiang et al. performed a randomized clinical trial [18], while the 15 others were prospective studies.

Jiang et al. had no baseline DCE-CT scan [18], while all others performed at least one baseline DCE-CT scan to compare to scans post-treatment. Two studies by Ng et al. had two baseline DCE-CT scans [16,17].

In 12 studies, patients with non-small cell lung cancer (NSCLC) were included. Fraioli et al. included patients specifically with adenocarcinoma [20]. Sudarski et al. included patients with NSCLC and patients with small cell lung cancer (SCLC) [21], Zhao et al. included patients with all types of lung cancer [22], and Hegenscheid et al. included patients with pulmonary metastases [23]. 

The CT scanners used in 15 articles varied from 8 to 320 slices. Six of these scanners were less than 64-slice scanners. In one study by Zhao et al. the model of scanner was not available [22]. 

### 3.2. Systemic Chemotherapy with or without Anti-Angiogenic Drug (Seven Studies)

Data presented in Table 1 vary in time interval from treatment start to the first follow-up DCE-CT scan, with time between these two events ranging from 21 to 90 days.

The DCE-CT scans were executed with different scan protocols and scanners. Patients received between 40 mL and 108 mL of intravenous non-ionic contrast media—none of which were weight dependent—and with different flow rates and intervals. In four studies, tube voltage ranged from 80 kVp to 120 kVp and tube current ranged from 36 mAs to 120 mAs [20,21,22,24]. In three studies, scan parameters were not available [25,26,27].

In four of the seven studies there was a significant decrease in permeability between baseline DCE-CT scans and DCE-CT scans performed after treatment start [20,21,24,25]. Zhang et al. showed a decrease in permeability, but did not test for significance between baseline scan and follow-up, instead testing for a significant difference in decreased permeability between two different treatment methods [27]. Two studies found no significant change in permeability [22,26].

In two studies, blood flow decreased significantly after treatment [20,24], three studies showed no significant changes [21,22,26], and Tacelli et al. did not measure blood flow [25]. Zhang et al. found a decrease in blood flow but tested for significance between two different treatment methods [27].

Blood volume decreased significantly after treatment in four studies. Of these four studies, a study by Fraioli et al. showed significant decrease for all included patients [20], a study by Tacelli et al. showed a significantly greater decrease in responders than non-responders in patients treated with chemotherapy and anti-angiogenic drug [25], a study by Wang et al. showed a significant decrease in responders only [26], and one study by Zhao et al. showed a significant decrease of blood volume in responders and a significant increase of blood volume in non-responders [22]. Fraioli et al. showed a significant difference in blood volume between subtypes of NSCLC [24], while Sudarski et al. showed a significant difference in blood volume between NSCLC and SCLC [21]. Zhang et al. found a decrease in blood volume, but tested for a significant difference in decreased blood volume between two different treatment methods instead [27]. Wang et al. also found that a difference in blood volume was a significant indicator of progression-free survival, and showed that blood volume and progression-free survival were significantly and inversely correlated [26]. 

Three studies divided patients into treatment groups of chemotherapy combined with anti-angiogenic drugs or chemotherapy alone [24,25,26]. Tacelli et al. found a significant decrease in permeability and blood volume in the group of patients who were treated with combined chemotherapy and anti-angiogenic drug, and no significant changes in the group treated with chemotherapy alone [25]. Wang et al. found significantly longer progression-free survival in responders treated with combined chemotherapy and anti-angiogenic drugs than responders treated with chemotherapy alone [26]. The same study showed a significant decrease in blood volume after treatment in responders in the combined arm (chemotherapy combined with antiangiogenic drugs), but no significant changes in the single arm (chemotherapy). Furthermore, a significant inverse correlation between blood volume and progression-free survival was found only in the combined arm. Fraioli et al. did not test the different treatments against each other [24]. 

### 3.3. Radiotherapy (3 Studies)

All three articles concerning radiotherapy were written by the same authors, Ng et al., and were performed with uniform scan protocols. The patients all received non-ionic contrast media with the same intervals, flowrate, and amount (108 mL). In two studies, the tube current was 120 mAs [15,16] and in one study a tube current of 60 mAs was used [17]. All three studies scanned at 80 kVp and had first follow-up scans one week after baseline scans. All patients included in the three studies were treated with palliative fractionated radiotherapy.

One study showed that permeability increased significantly in tumor rim after a second fraction of radiotherapy [16]. Another study showed that permeability increased significantly in six out of eight patients after radiotherapy, and the changes were greater at the rim of tumor, as opposed to the center [15]. One study did not measure permeability [17].

All studies measured blood volume. Two studies showed a significant increase after treatment when whole tumor was evaluated [16,17]. One study showed a decrease in blood volume, after treatment of the anti-vascular drug (CA4P) [15].

Patients in one study were given CA4P one week after second fraction radiotherapy [15]. This study showed a significant decrease in blood volume 4–72 h after patients received the anti-vascular drug, and significantly greater decrease of the tumor rim than center. The increase of permeability after radiotherapy, before CA4P, correlated with the decrease of blood volume in tumor rim after CA4P. 

### 3.4. Chemoradiotherapy (One Study)

Wang et al. did not distinguish between patients treated with chemotherapy, radiotherapy, or concurrent chemoradiotherapy [28]. Based on RECIST, responders had a significantly higher blood flow than non-responders prior to treatment. Patients who underwent a second DCE-CT scan after treatment were divided into two groups according to changes of permeability. The group with a decrease in permeability had a significantly longer median progression-free survival period and longer median overall survival period than the group with an increase in permeability.

### 3.5. Others (Five Studies)

None of the patients in this group were treated with systemic chemotherapy or radiotherapy. Three studies used target therapy of RHES, anti-EGFR, or anti-angiogenic drugs. Hegenschied et al. used laser-induced thermotherapy [23] and Li et al. used intra-arterial infused chemotherapy [29].

Jiang et al. performed no baseline scan [18]. First follow-up scans varied from one day to three weeks after treatment. Li et al. only performed baseline scans [29]. Scans were executed with tube voltage ranging from 80 to 120 kVp and tube current of 50–115 mAs. In four studies, patients received between 40 and 50 mL non-ionic contrast media, while Qiao et al. dosed contrast media according to weight [30]. 

Li et al. used dual input to distinguish between bronchial and pulmonary blood flow [29]. The remaining four articles placed a single region-of-interest (ROI) in aorta and used a single input to extract perfusion data.

Hegenschied et al. showed a significant increase in mean transit time and a significant decrease in blood flow, blood volume, and permeability one day after treatment [23]. 

The three studies on target therapy treatment all showed an effect on blood flow. For patients treated with anti-angiogenic drugs, Jiang et al. showed a significant increase from day 1 to day 5, and then a significant decrease from day 5 to day 10 [18]. Lind et al. showed a significant decrease in blood flow 3–6 weeks after treatment in patients treated with anti-angiogenic drugs and anti-EGFR drugs [19]. They also found significantly lower blood flow in responders than in non-responders 3–6 weeks after treatment. Qiao et al. also found a significant decrease in blood flow in responders treated with anti-EGFR [30]. The same study showed that patients with a decrease in blood flow had a significantly longer median progression-free survival. 

Li et al. measured bronchial flow, and showed that responders had a significantly higher bronchial flow than non-responders before treatment [29]. They also showed that bronchial flow is a significant prognostic factor for progression-free survival and overall survival.

### 3.6. Bias and Applicability 

The studies included in this analysis were evaluated on risk of bias and applicability by two authors (L.S.S. and C.A.L.) according to QUADAS-2. Results of the QUADAS-2 test are shown in Table 3.

## 4. Discussion

Currently, there is no consensus on methods of measurements of perfusion using DCE-CT. The heterogeneity in setup and scan parameters makes it difficult to compare the studies presented in this paper. In studies measuring permeability before and after chemotherapy, four of seven showed a significant decrease in permeability after treatment [20,21,22,24,25,26,27]. Only two studies [15,16] assessed changes in permeability after radiotherapy and both found an increase, however this finding would not necessarily reflect treatment response, but rather vessel damage and inflammation caused by the radiotherapy [31]. This systematic review indicates that cancer treatments, except radiotherapy, have an effect on permeability, resulting in its decrease. Tumors are often hyperpermeable, due to high density dysplastic vessels with abnormalities in vessel wall structure and large pores [32]. Hence, a decrease in permeability would suggest a normalization of the blood vessels in the tumor indicating treatment response. Permeability seems to be a promising perfusion value to estimate early treatment response. 

Studies where patients were treated with anti-angiogenic drugs generally showed decrease in blood flow. Of the five studies that measured blood flow in patients who were treated with anti-angiogenic drugs, four showed a significant decrease in blood flow [19,20,24,26,27]. Because of tumor heterogeneity, blood flow is more complex and can vary considerably within a tumor. In parts of tumor with angiogenesis and where growth is active, such as the periphery of tumor, blood flow increases. However, in regions with high interstitial pressure, the capillaries will be compressed causing blood flow to decrease. This often results in areas of tissue hypoxia and necrosis [33]. 

Four out of five studies that assessed the correlation between DCE-CT values and progression-free survival found that a decrease in permeability [28] or blood flow [19,29,30] corresponded to longer progression-free survival. Yao et al. found similar results for blood flow in patients with advanced carcinoid tumor treated with anti-angiogenic drugs [34]. Bisdas et al. found blood flow to be a predicator of progression-free survival in patients with oropharynx squamous cell carcinoma [35]. Both studies however were small and included 44 and 19 patients, respectively. Hence, further investigation is needed to draw any conclusions between blood flow and survival outcome.

Only one of the included studies was a randomized control trial [18]; the remaining were prospective studies, which affect the level of evidence. In 12 of the 16 included studies, the reference standard was subject to high risk of bias and concerns of applicability. All but four studies used RECIST as a reference standard, and as there are concerns as to whether this tool for evaluating treatment response is the most accurate, especially in patients treated with anti-angiogenic drugs, the studies were disposed to high risk of bias.

Different vendors use different models to calculate perfusion values. Depending on which scanner is available, the kinetic model is predefined and they each have their pros and cons. The most widely used kinetic models are deconvolution and compartmental [12,36]. The compartmental method is calculated from the maximum slope of attenuation either as a single compartment or double-compartment. Single compartment analyses consider the intravascular and the extravascular spaces as a single compartment [36]. During the first pass of contrast media (45–60 s after injection), most contrast media remains in the intravascular space, allowing the assumption of single compartment [36]. The studies which made use of the single compartment method were able to determine blood flow within a short overall length of scan time (≥45 s), which is a great advantage, especially when assessing lung tumors which are prone to breathing artifacts. The disadvantage is that this method is very sensitive to image noise and not able to measure permeability, which seems to be an important indicator of treatment response. The double-compartment is, however, able to measure permeability. This is done based on Patlak analysis which was performed in the majority of included studies [15,16,17,20,22,24,25]. This makes the assumption that there is no backflux of contrast media from the interstitial space to the intravascular space. For this assumption to be acceptable several conditions have to be met, and even then it is only valid for the initial 2 min [32,33]. The compartment models require a high flowrate of contrast media (>7 mL/s) and is very susceptible to noise, which is why scans are often executed with higher tube current [36].

Two of the included studies used the deconvolution method [21,23]. This method uses time-density curves to calculate impulse residue function [32]. The deconvolution method tolerates greater images’ noise, which allows lower tube current and higher temporal resolution due to the higher number of images. This, however, makes the deconvolution method predisposed to motion artifacts [37] and patients are exposed to higher overall radiation dose [32]. Deconvolution method is appropriate for measuring lower levels of perfusion (<20 mL/min/100 mL), which is beneficial in evaluation of treatment response [36].

Very little data are available on direct comparison of these analysis methods, but preliminary investigations by Griffiths et al. have shown good correlation between the two methods in lung and spleen lesions, however the slope method showed consistently lower perfusion values. Hence, comparison of the two analytical methods should be done with caution [12].

The study by Li et al. was the only study to use a dual input method to determine the perfusion values [29]. Lung tumors usually have a dual vascular supply, from both bronchial and pulmonary arteries. Studies have shown that the perfusion values differ from tumor location and size [38,39]. 

The clinical potential of DCE-CT is the possibility of early detection of perfusion changes corresponding to early response. The earliest response detected in the included studies were three weeks after start of chemotherapy [22,25] which would be at a time where a decrease in size could be difficult to measure with RECIST criteria. According to RECIST, a frequency of tumor re-evaluation every 6–8 weeks is reasonable [6]. The potential to assess treatment response at an earlier time would enable the possibility to quickly change treatment for nonresponding patients, which would benefit patients and save money on unnecessary treatments.

Positron emission tomography (PET)/CT is an alternative functional imaging tool used to evaluate tumor response. While DCE-CT measures tumor vascularity, PET/CT can be used to measure tumor metabolism and tumor hypoxia using fludeoxyglucose (FDG)-PET and hypoxia PET. According to Harders et al. ^18^F-FDG PET/CT is more validated and therefore preferred when it comes to diagnostic and staging of lung tumors. Therapy evaluation lacking in standardization for both modalities, but DCE-CT has shown great potential in patients treated with anti-angiogenic drugs in multiple smaller studies [40]. Since PET and DCE-CT seem to measure different aspects of tumors, few studies have investigated the correlation between these. However, Kim et al. found a significant positive correlation between parameters acquired from ^18^F-FDG PET/CT and DCE-CT parameters in patients with liver metastasis [41]. In patients with NSCLC, Miles et al. found a significant correlation between metabolism and blood flow derived from ^18^F-FDG PET/CT and DCE-CT, respectively, but only for tumors less than 4.5 cm^2^ [42]. By contrast, van Elmpt et al. found no correlation between tumor metabolism and tumor vascularity on either population or subvolume level in patients diagnosed with NSCLC. Still, they did find a negative correlation between perfusion values, such as blood volume and blood flow, and hypoxia parameters [43]. Compared to PET/CT, DCE-CT has the advantage of being more available due to the widespread use of CT scanners. Furthermore, it is a faster and cheaper examination.

Another functional imaging tool which is generally more applied than DCE-CT is DCE-MRI, possibly because radiation dose does not need to be taken into count. Except from the difference in modalities, the overall method of the DCE-MRI is similar to that of DCE-CT, with some major differences. The contrast agent used in DCE-MRI is gadolinium and the most commonly used parameters are area under the contrast-agent-concentration time curve at 60 s (IAUC_60_) and volume transfer contrast (K_trans_). DCE-MRI is widely used in drug development, especially in development of vascular-targeting agents. O’Connor et al. demonstrates that even though DCE-MRI is able to monitor changes in vascularity, especially K_trans_, the lack of standardization causes the method to still be considered controversial. They point out that changes in DCE-MRI parameters do not guarantee survival benefits and hence changes in vascularity are necessary, but not sufficient, to evaluate efficiency of vascular-targeting agents [44].

The main limitation of this review is the number and heterogeneity of the included studies. Some of the factors that make generalization uncertain are the differences in hardware, e.g., different vendors and scanner models, and the difference in scan protocols. Due to the fact that DCE-CTs are generally performed with highly various scan parameters, it is not possible to make a direct comparison of perfusions values, as they are affected by scan parameters such as tube current, tube voltage, scan duration, and contrast injection. Further complicating comparison is the fact that no consensus has yet been reached as to which analytic method is preferred to measure treatment response, and no standard of time is determined for when to perform follow-up scans to detect early changes in tumor. DCE-CT as a tool to evaluate treatment response is relatively new, and thus larger randomized studies are needed to establish standardizations.

Some of the limitations DCE-CT is restricted to is the number of detector rows of the scanner. Only three of the included studies used a CT scanner with more than 64 slices [21,26,29]. Hence, most studies were limited to a scan range of 4 cm or less, which disables whole tumor evaluation of larger tumors. DCE-CT of the lung is prone to different artifacts. Due to the high concentration of contrast media in the great vessels and the heart chambers, there are risks of beam-hardening, which should try to be avoided because it can affect perfusion values significantly [10]. Another artifact seen in DCE-CT of the lungs is respiratory motion. Most of the included studies worked with breath-hold or shallow breathing. Breath-hold is not appropriate for scans longer than 45 s as many patients tend to slowly exhale during the scan, causing motion artifacts [32]. Therefore, motion correction software is often applied [40]. Motion artifacts have a big influence on reproducibility. Lee et al. found that reproducibility improved with whole tumor coverage and motion correction [45]. Other studies have shown generally acceptable [46] to good reproducibility [47] of perfusion values in patients with colorectal and rectal cancer, respectively. Goh et al. found that reproducibility in colorectal cancer was superior to that of skeletal muscle [48]. Few studies have investigated reproducibility in DCE-CT in lung cancer, probably due to the risk of radiation. Ng et al. demonstrated variability in measurements of blood flow and permeability at best 11.6% and 30.2%. They concluded that reproducibility of perfusion values in lung tumors are very much affected by motion and duration of data acquisition [49].

In conclusion, DCE-CT is a potential tool of evaluating tumor response in patients with lung cancer. Permeability and blood flow seem to be important perfusion values regarding assessment of early treatment response. However, the heterogeneity of scan protocols, scan parameters and time to follow-up, in the included studies complicates a comparison of the utilized technique. Hence, more collaborative research and more standardized protocols are required.

## Figures and Tables

**Table 1:** Overview of included studies—Systemic chemotherapy.

Systemic Chemotherapy (+/− Anti-Angiogenic Drug)														
Author, Year	Study Design	Patients	Diagnosis	Scan Parameters			Kinetic Model	Aim	Treatment	Perfusion Scan	DCE-CT* Values	Gold Standard	Results	Conclusion
				Slice	kVp mAs	Contrast								
Fraioli et al. 2011 [20]	Prospective	45	Lung adenocarcinoma	64	100 kVp 120 mAs	90 mL	Two-compartmental (Patlak)	To determine if DCE-CT* enables evaluation of the effects of chemotherapy combined with anti-angiogenetic drug and to determine if changes in CT correlate with RECIST*.	Chemotherapy combined with anti-angiogenic drug	Baseline, 40 (*n* = 45) and 90 days after treatment (*n* = 14)	BF* BV* TTP* PS*	RECIST*	Significant decrease from baseline to follow-up in BF* (*p* = 0.018) and PS* (*p* = 0.013).	DCE-CT* may allow evaluation of lung cancer angiogenesis demonstrating alterations in vascularity following treatment.
Fraioli et al. 2013 [24]	Prospective	50	NSCLC*	64	100 kVp 120 mAs	90 mL	Two-compartmental (Patlak)	To determine if DCE-CT* can be used to evaluate the effects of chemotherapy and anti-angiogenic treatment in patients with NSCLC* and whether DCE-CT* and RECIST* before and after therapy correlate.	Non squamous carcinoma (*n* = 36): Chemotherapy combined with anti-angiogenic drug Squamous cell carcinoma (*n* = 14): Chemotherapy	Baseline and 90 days after treatment	BF* BV* TTP* PS*	RECIST*	Significant decrease from baseline to follow-up in BF* (*p* = 0.001) and PS* (*p* = 0.001) Significant difference in BV* between the subtypes. RECIST* classifications showed a difference in BF* (*p* = 0.001), BV* (*p* = 0.008) and TTP* (*p* = 0.007) between responder and non-responder.	Difference in DCE-CT* parameters between subtypes of lung cancer before and after treatment may play an important role in assessing early treatment response.
Sudarski et al. 2015 [21]	Prospective	100	NSCLC* (*n* = 84) and SCLC* (*n* = 16)	128	80 kVp 36 mAs	50 mL	Deconvolution	To compare DCE-CT* parameters with RECIST* for prediction of therapy response and OS* in NSCLC* and SCLC* patients treated with conventional chemotherapy.	Chemotherapy	Baseline and after treatment (within median of 44 days)	BF* BV* MTT* PS*	RECIST*	Significant decrease from baseline to follow-up in PS* (*p* = 0.009) and MTT* (*p* = 0.007). Significant higher BV* (*p* = 0.002), MTT* (*p* = 0.009) and PS* (*p* = 0.003) in NSCLC* patients than SCLC* patients at follow-up. A significant decrease in MTT* (*p* = 0.0005) in SCLC* patients between baseline and follow-up.	DCE-CT* parameters differ between NSCLC* and SCLC*. DCE-CT* values do not relate to RECIST* and do not improve prediction of OS* in patients treated with conventional chemotherapy.
Tacelli et al. 2013 [25]	Prospective	40	NSCLC*	64	N/A*	108 mL	Two-compartmental (Patlak)	Can DCE-CT* depict early perfusion changes in lung cancer treated by anti-angiogenic drugs, allowing prediction of response	Group 1 (*n* = 17): Chemotherapy combined with anti-angiogenic drug Group 2 (*n* = 23): Chemotherapy	Baseline, 21 days (*n* = 40), 63 days (*n* = 34) and 126 days (*n* = 26) after treatment start	TVV ^1^ TEF ^2^	RECIST*	Group 1: Significant decrease in TVV ^1^ and TEF ^2^ between baseline and all three follow-ups (*p* < 0.05). Significant difference in TVV ^1^ between responders and non-responders measured at baseline and first follow-up (*p* = 0.0128).	DCE-CT* can depict early changes in tumor vasculature in NSCLC* patients treated with conventional chemotherapy combined with anti-angiogenic drug.
Wang et al. 2013 [26]	Prospective	74	NSCLC*	128	N/A*	100 mL	N/A*	Tumor blood volume in DCE-CT* and CEC* might predict the status of angiogenesis. The present study aimed to validate their representation as feasible predictors in non-small-cell lung carcinoma.	Group 1 (*n* = 38): Chemotherapy combined with anti-angiogenic drug Group 2 (*n* = 36): Chemotherapy	Baseline and every 6–8 weeks during treatment	BF* BV* MTT* PS*	RECIST*	Group 1: PFS* was significantly longer (*p* = 0.034) in the CBR ^3^ group at follow-up. Significant decrease in BV* (*p* = 0.034) at follow-up compared to baseline in non-PD*. ΔBV* is a significant (*p* = 0.019) indicator of PFS*.	BV* can predict anti-angiogenic efficacy and is in combination with CEC* more reliably than plain or enhanced CT alone.
Zhang et al. 2015 [27]	Prospective	76	NSCLC*	64	N/A*	40 mL	N/A*	To study the effectiveness of an anti-angiogenic drug combined with chemotherapy in treating advanced NSCLC* and to evaluate outcome by DCE-CT* imaging.	Group 1 (*n* = 36): Anti-angiogenic drug administered from day 1 and combined with chemotherapy from day 5 Group 2 (*n* = 40): Anti-angiogenic drug combined with chemotherapy from the first day	Before chemotherapy start and 45–50 days later	BF* BV* MTT* PS*	RECIST*	Group 1: Significantly fewer patients with PD* (*p* = 0.039) compared to group 2. Significantly higher RR ^4^ (*p* = 0.032) and CBR ^5^ *(p = 0.0045)*. Significantly difference in decrease of BF* (*p* = 0.034), BV* (*p* = 0.019), and PS* (*p* = 0.006) between group 1 and 2. Significantly difference in increase of MTT* (*p* = 0.0124) between the two groups.	The study suggests that an anti-angiogenic drug administrated four days before chemotherapy is better than chemotherapy combined with the anti-angiogenic from the first day. DCE-CT* could be a reasonable method for evaluating patients after treatment.
Zhao et al. 2014 [22]	Prospective	25	Lung cancer	N/A*	120 kVp 100 mAs	40 mL	Two-compartmental (Patlak)	To observe the changes in DCE-CT* parameters of patients with early stage lung cancer before and after chemotherapy	Chemotherapy	Baseline and 21–25 days later	BF* BV* PS* PBV*	RECIST*	Patients were divided into responders (*n* = 15) and non-responders (*n* = 10) based on a regular CT performed before treatment and 100 days later. Remission group: Significant decrease in BV* (*p* = 0.023) and PBV* (*p* = 0.005) after treatment. Non-remission group: Significant increase in BV* (*p* = 0.016) and PBV* (*p* = 0.036) after treatment.	Increase in PBV* in the early stage after chemotherapy indicates that patients are not sensitive to treatment. Decrease in PBV* indicates the opposite. Change of PBV* is valuable for assessment of effects of chemotherapy.

* DCE-CT = Dynamic Contrast-Enhanced CT, RECIST = Response evaluation criteria in solid tumors, BF = Blood flow, BV = Blood volume, TTP = Time to Peak, PS = Permeability surface area product, NSCLC = Non-small cell lung cancer, PFS = Progression-free survival, OS = Overall survival, SCLC = Small cell lung cancer, MTT = Mean Transit Time, N/A = Not available, CEC = Circulating endothelial cells, PD = Progressive disease, PBV = Patlak blood volume; ^1^ TVV = Total Vascular Volume (TVV = BV × VPCT); VPCT = Total volume of voxels included in the analysis; ^2^ TEF = Total Extravascular Flow (TEF = K-trans × VPCT); VPCT = Total volume of voxels included in the analysis; ^3^ CBR = Clinical benefit rate = (CR + PR + SD)/total × 100%; ^4^ RR = Response Rate (CR+PR); ^5^ CBR = Clinical Beneficial Rate (CR + PR + SD).

**Table 2:** Overview of included studies grouped by the remaining treatments.

Author, Year	Study Design	Patients	Diagnosis	Scan Parameters			Kinetic Model	Aim	Treatment	Perfusion Scan	DCE-CT* Values	Gold Standard	Results	Conclusion
				Slice	kVp mAs	Contrast								
**Radiotherapy**														
Ng et al. 2007 [16]	Prospective	16	NSCLC*	16	80 kVp 120 mAs	108 mL	Two-compartmental (Patlak)	To assess the in vivo acute vascular effects of fractionated radiotherapy for human NSCLC* using DCE-CT*.	Palliative fractionated radiotherapy	Baseline, 1 week (*n* = 16), 2 weeks (*n* = 8) and 3 weeks (*n* = 6) later	BV* PS*	N/A*	BV* increased significantly when comparing first (*p* = 0.025), second (*p* = 0.018) and third (*p* = 0.002) follow-up with baseline. After second and third follow-up an increase in both BV* (*p* = 0.034 & *p* = 0.0012) and PS* (*p* = 0.022 & *p* = 0.0048) were found in the rim of tumor.	Radiation increases BV* and PS* in NSCLC* and these vascular effects are more pronounced at the rim compared to center
Ng et al. 2007 [15]	Prospective	8	NSCLC*	16	80 kVp 120 mAs	108 mL	Two-compartmental (Patlak)	To study the tumor vascular effects of radiotherapy and subsequent administration of vascular disrupting agent (CA4P) in patients with advanced NSCLC* using DCE-CT*.	Hypo-fractionated palliative radiotherapy and CA4P	Baseline, 1 week later before CA4P, 4h after CA4P and 72 h after CA4P	BV* PS*	N/A*	BV* decreased significantly four hours after CA4P (*p* = 0.029) which sustained to 72 h (*p* = 0.025). PS* increased significantly in tumor rim after second fraction (*p* = 0.0073). Four hours after CA4P, BV* decreased significantly more in tumor rim (*p* = 0.035) than tumor center (*p* = 0.0077). This sustained to 72 h (*p* = 0.014 & *p* = 0.012, respectively). Increase in PS* after radiotherapy correlated to reduction in BV* after CA4P at tumor rim (*p* = 0.020).	Radiotherapy enhances the tumor anti-vascular activity of CA4P in human non-small-cell lung cancer, resulting in sustained tumor vascular shutdown.
Ng et al. 2010 [17]	Prospective	15	NSCLC*	16	80 kVp 60 mAs	108 mL	Two-compartmental (Patlak)	To assess the distribution of BV* in lung tumor, and to establish if whole tumor assessment is more representative of the vascular effect of radiotherapy than conventional single level.	Palliative fractionated radiotherapy	Baseline and 1 week later	BV*	N/A*	BV* increased significant (*p* = 0.049) after radiotherapy using whole tumor evaluation but not with single tumor evaluation.	Whole tumor DCE-CT* may be a better predictor of vascular changes following therapy compared to conventional single tumor level evaluations.
**Chemoradiotherapy**														
Wang et al. 2009 [28]	Prospective	35	NSCLC*	16 or 8	120 kVp 50 mAs	50 mL	N/A*	To evaluate changes in tumor perfusion values after chemo-radiation therapy, and to investigate the feasibility of DCE-CT* for prediction of early tumor response and prognosis of NSCLC*.	Chemotherapy, radiation therapy or concurrent chemoradiotherapy	Baseline (*n* = 35) and after two cycle chemotherapy or before the end of radiotherapy (*n* = 22)	BF* BV* MTT* PS*	RECIST*	BF* at baseline were significantly higher (*p* = 0.023) in responders than non-responders. The follow-up patients were divided into two groups due to changes in PS* after treatment. The group with decrease of PS* (*n* *=* 11) had a significant longer median PFS* (*p* < 0.001) and median OS* (*p* = 0.004) than the group with increase in PS* (*n* = 11).	NSCLC* with high perfusion is relatively sensitive to chemo-radiation therapy. DCE-CT* is useful in predicting early tumor response and the prognosis of NSCLC* after treatment.
**Others**														
Hegenscheid et al. 2009 [23]	Prospective	12	Pulmonary metastases *(n=22)*	8	120 kVp 115 mAs	40 mL	Deconvolution	To use DCE-CT* to monitor early vascular changes in tumor perfusion after laser-induced thermotherapy (LITT) and to determine whether any of the perfusion parameters would predict technical success after therapy.	LITT	Baseline, 1 day and 4–6 weeks after treatment	BF* BV* MTT* PS*	RECIST*	Significant decrease in BV* (*p* < 0.001), BF* (*p* < 0.001) and PS* (*p* < 0.001) 1 day after treatment compared to baseline. Significant increase in MTT* (*p* < 0.498) 1 day after treatment compared to baseline.	DCE-CT* can be useful for assessing tumor vascularity and changes in perfusion after LITT. Significant reduction in BV*, BF* and PS* 1 day after treatment could indicate technical effectiveness.
Jiang et al. 2012 [18]	Randomized clinical trial	15	NSCLC* in patients who were hypoxia-positive indicated by SPECT/CT	16	120 kVp 50–80 mAs	40 mL	N/A*	To confirm that RHES* has a “time window” of vascular normalization also in human tumors.	Research group (*n* = 10): RHES* for 10 days Control group (*n* = 5): No treatment	1, 5 and 10 days after treatment	BF* BV* MTT* PS*	N/A*	Research group: Significant increase in BF* (*p* < 0.01*)* from day 1 to day 5. Significant decrease in BF* (*p* < 0.01) from day 5 to day 10. Significant decrease in PS* (*p* < 0.01) from day 1 to day 5. Increase trend in PS* (*p* = 0.69) from day 5 to day 10. BV* in the research group were significant higher (*p* = 0.000) on day 5, than in the control group. PS* in the research group were significant lower (*p* = 0.001) on day 5, than in the control group.	The study confirms that there is a RHES* “time window” of vascular normalization in human body.
Li et al. 2014 [29]	Prospective	42	NSCLC*	320	80 kVp 40 mAs	50 mL	Single-compartmental (Maximum slope) Dual input (Aorta & Pulmonary artery trunk)	To evaluate tumor perfusion using dual-input DCE-CT* in advanced NSCLC* and to determine whether the effect of multiarterial infusion chemotherapy can be predicted in light of perfusion parameters.	Intra-arterial chemotherapy	Baseline	Bronchial flow Pulmonary flow Perfusion Index	RECIST*	At baseline responders had a significant higher bronchial flow (*p* = 0.02) compared to non-responders. Bronchial flow is a significant prognostic factor for PFS* (*p* = 0.01) and OS* (*p* = 0.02).	Dual-input DCE-CT* may be useful in predicting effect of treatment. Tumors with high bronchial flow may have a good response to treatment. Bronchial flow is a significant prognostic factor for PFS* and OS*.
Lind et al. 2010 [19]	Prospective	23	NSCLC*	64	100 kVp 80 mAs	50 mL	Single-compartmental (Maximum slope)	To investigate the feasibility of DCE-CT* in NSCLC* patients receiving anti-angiogenic and anti-EGFR* treatment, and to correlate tumor BF to treatment outcome.	Anti-angiogenic drug & Anti-EGFR*	Baseline, 3 (*n* = 23) and 6 weeks (*n* = 19) after treatment	BF*	RECIST* Crabb	Significant decrease in BF* from baseline to week 3 (*p* < 0.001) and week 6 (*p* < 0.001), respectively. Responders had a significant lower BF* than non-responders at 3 weeks (*p* = 0.03) and 6 weeks (*p* = 0.04). Baseline BF* was significantly lower (*p* = 0.012) in pt. who developed extensive cavitations than those who did not. Patients with a decrease in BF* larger than the median at week 6 tended to have a longer PFS (*p* = 0.06)	DCE-CT* appears to be feasible in patients with NSCLC*. This technique demonstrated a decrease in tumor BF* following anti-angiogenic and anti-EGFR* therapy.
Qiao et al. 2015 [30]	Prospective	20	NSCLC*	64	100 kVp 200 mA	1.5 mL/kg	Single-compartmental (Maximum slope)	To study the feasibility and clinical value of DCE-CT for early evaluation of targeted therapy in NSCLC*.	Anti-EGFR*	Baseline and 7 days after treatment	BF* PH* TTP* M/A*	RECIST*	Patients who were classified as PR* had a significant decrease in BF* (0.0225) after treatment. PD* had significant increase in M/A* (0.0443) and BF* (0.0268) after treatment. Patients were divided into groups of increase in BF* and decrease in BF*. The group with increase in BF* had a median PFS* of 6 weeks. The group with decrease in BF* had a median PFS* of 54 weeks. (*p* = 0.0001)	DCE-CT* permits early assessment of targeted therapy efficacy. Increased BF* indicates that tumor do not respond to treatment, whereas decreased BF* suggests that treatment is effective.

* DCE-CT = Dynamic Contrast-Enhanced CT, NSCLC = Non-small cell lung cancer, BV = Blood volume, PS = Permeability surface area product, N/A = Not available, BF = Blood flow, MTT = Mean Transit Time, RECIST = Response evaluation criteria in solid tumors, PFS = Progression-free survival, OS = Overall survival, RHES = Recombinant human endostatin, EGFR = Epidermal growth factor receptor, PH = Peak Height, TTP = Time to Peak, M/A = tumor mass-aortic peak height ratio, PR = Partial response, PD = Progressive disease.

**Table 3:** Evaluation of risk of bias and applicability of studies included in the analysis.

Study	Risk of Bias				Applicability Concerns		
	Patient Selection	Index Test	Reference Standard	Flow and Timing	Patient Selection	Index Test	Reference Standard
Fraioli et al. 2011 [20]	☺	☹	☹	☺	☺	☺	☹
Fraioli et al. 2013 [24]	☺	?	☹	☺	☺	☺	☹
Sudarski et al. 2015 [21]	☺	?	☹	☺	☺	☺	☹
Tacelli et al. 2013 [25]	☺	?	☹	☺	☺	☺	☹
Wang et al. 2013 [26]	☺	?	☹	☺	☹	☺	☹
Zhang et al. 2015 [27]	☺	☹	☹	☺	☺	☺	☹
Zhao et al. 2014 [22]	☺	☹	☹	☺	☺	☺	☹
Ng et al. 2007 [16]	☺	?	?	?	☺	☺	?
Ng et al. 2007 [15]	☺	?	?	?	☺	☺	?
Ng et al. 2010 [17]	☺	?	?	?	☺	☺	?
Wang et al. 2009 [28]	☺	☹	☹	☺	☺	☺	☹
Hegenscheid et al. 2009 [23]	☺	☺	☹	☺	☺	☺	☹
Jiang et al. 2012 [18]	☺	?	?	?	☺	☺	?
Li et al. 2014 [29]	☺	☺	☹	☺	☺	☺	☹
Lind et al. 2010 [19]	☺	☺	☹	☺	☺	☺	☹
Qiao et al. 2015 [30]	☺	☺	☹	☺	☺	☺	☹

☺ Low Risk; ☹ High Risk; ? Unclear Risk.

## Abbreviations

The following abbreviations are used in this manuscript:

DCE-CT	Dynamic Contrast- Enhanced Computed Tomography
PRISMA	Preferred Reporting Items for Systematic Reviews and Meta-Analyses
QUADAS	Quality Assessment of Diagnostic Accuracy Studies
RECIST	Response Evaluation Criteria in Solid Tumors
MeSH	Medical Subject Headings
RHES	Recombinant Human Endostatin
EGFR	Epidermal Growth Factor Receptor
NSCLC	Non-Small Cell Lung Cancer
SCLC	Small Cell Lung Cancer
CA4P	Combretastatin A4 Phosphate
PET	Positron Emission Tomography
